# Supplementary material for: In support of 2D:4D: More data exploring its conflicting results on handedness, sexual orientation and sex differences
Source: PLoS One. 2023 Aug 22;18(8):e0280514. doi: 10.1371/journal.pone.0280514 (PMC10443882; doi:10.1371/journal.pone.0280514)
Supplement: Attachment — Submitted filename: Reviewer Report-PONE-D-22-03299.docx [file pone.0280514.s002.docx]

**The case against 2D:4D: more data about its conflicting results on handedness, sexual orientation and sex differences.**

**Manuscript ID: PONE-D-22-03299**

Thank you for the opportunity to review this manuscript. The authors sought to test whether sexual dimorphism in the 2D:4D is due to allometric or isometric changes in the human fingers and whether the 2D:4D can represent prenatal hormone exposure. They further sought to determine whether the 2D:4D has an impact on sexual orientation and handedness in both adult males and females. It was observed that the 2D:4D may be used as an index of prenatal hormone exposure, but with caution. Moreover, the 2D:4D was associated with sexual orientation in only males but no association with handedness was detected. The manuscript is well written and well discussed. I will recommend its publication if the authors can address these minor revisions:

**Methods/results**

1. Line 282-284: “Based on the data obtained in the population for which we had both measures, we decided to consider those subjects with values greater than 0.7 as heterosexual, those with values below 0.3 as homosexual, and those with values between 0.3 and 0.7 as bisexual”. What was the scientific basis for your decision?
2. Line 334-335:” The slope is not significantly different from 0 except in the regression fitted for the right-hand data for the men in the full sample”. Line 386: “We used t-test to test if the slopes are different from 0”. Line 398: “We used a t-test to test if the slopes differ from 1” Did you use a 1-tailed or 2-tailed test and at what confidence interval?
3. Table 1-6. You summarized the results as mean (SD) in Table 1 but mean (SEM) in Tables 4-6. What informed this decision and why were you not consistent?
4. Table 1: It might be useful to define the effect size in the table legend as; Cohens *d*: negligible (*d*<0.20), **small** (0.20≤*d*<0.50), ***medium*** (0.50≤*d*≤0.80) and **large** (*d*>0.80).
5. Table 5: The difference in the 2D:4D is traditionally determined as the right-left difference (Dr-l). a lower Dr-l may indicate increased exposure to prenatal androgens as lower righthand 2D:4D is associated with masculine traits. However, you used a left-right difference (Dl-r). Any reason for that?
6. Table 6: The Kruskal-Wallis test is nonparametric ANOVA. If the variables were not normally distributed why were they not summarized as median (IQR) but rather mean±SEM?
